# Identification of NOTCH-driven matrisome-associated genes as prognostic indicators of multiple myeloma patient survival

**DOI:** 10.1038/s41408-023-00907-6

**Published:** 2023-09-05

**Authors:** Daniela Simone Maichl, Julius Arthur Kirner, Susanne Beck, Wen-Hui Cheng, Melanie Krug, Martin Kuric, Carsten Patrick Ade, Thorsten Bischler, Franz Jakob, Dirk Hose, Anja Seckinger, Regina Ebert, Franziska Jundt

**Affiliations:** 1 Department of Internal Medicine II, University Hospital Würzburg, Oberdürrbacher Straße 6, 97080 Würzburg, Germany; 2 Institute of Pathology, University Hospital Heidelberg, Im Neuenheimer Feld 224, 69120 Heidelberg, Germany; 3 Bernhard-Heine-Center for Locomotion Research, Department of Musculoskeletal Tissue Regeneration, University of Würzburg, Friedrich-Bergius-Ring 15, 97076 Würzburg, Germany; 4 Department of Biochemistry and Molecular Biology, Biocenter, University of Würzburg, Am Hubland, 97074 Würzburg, Germany; 5 Core Unit Systems Medicine, University of Würzburg, Josef-Schneider-Str. 2, 97080 Würzburg, Germany; 6 Bernhard-Heine-Center for Locomotion Research, Orthopedic Department, University of Würzburg, Brettreichstrasse 11, 97074 Würzburg, Germany; 7 Department of Hematology and Immunology, Myeloma Center Brussels, Vrije Universiteit Brussel, Laarbeeklaan 103, 1090 Brusells, Belgium

**Keywords:** Cancer microenvironment, Myeloma

Dear Editor,

Multiple myeloma (MM) is a rarely curable plasma cell malignancy of the bone marrow (BM) that provides a permissive tumor microenvironment (TME), supporting tumor cell growth and dissemination and conferring therapy resistance. The TME contains cellular components, specifically stromal cells, osteoclasts, osteoblasts, osteocytes, endothelial and immune cells, and a non-cellular component, the extracellular matrix (ECM). In cancer, the ECM is an important determiner of cell fate and composition of the TME. Recent research has coined the term “matrisome” for the ensemble of genes encoding ECM proteins and ECM-associated proteins and defined gene sets for core matrisome (approximately 274 genes) and matrisome-associated genes (approximately 1027 genes), including secreted modifiers [1]. Specified cancer matrisomes regulate proliferation, migration, and survival [1]. Hence, changes in ECM composition, integrity, abundance, biomechanical properties, and related signal transduction contribute to tumor progression and outcome in patients [1]. In MM, the ECM bidirectionally interacts with MM cells and co-inhabitants of tumor cell/metastatic niches. Notably, expression levels of genes in MM cells involved in the interaction with the TME have been linked to better (*BMP6* [2]) or worse (*ANXA2*, *LGALS1* [3, 4]) survival of patients. We and others have shown that NOTCH signaling alters the TME through juxtacrine signaling between signal-sending cells such as MM or stromal cells expressing the ligands, and signal-receiving cells expressing the receptors [5, 6]. Whether deregulated NOTCH signaling in MM cells controls the expression of genes that dysregulate ECM composition in the BM niche and have prognostic significance, is unknown. Here, we correlated transcriptome profiles of NOTCH-depleted MM cells with recently published matrisome libraries, to identify NOTCH-regulated genes that belong to the matrisome and are related to patient survival.

**Fig. 1:**
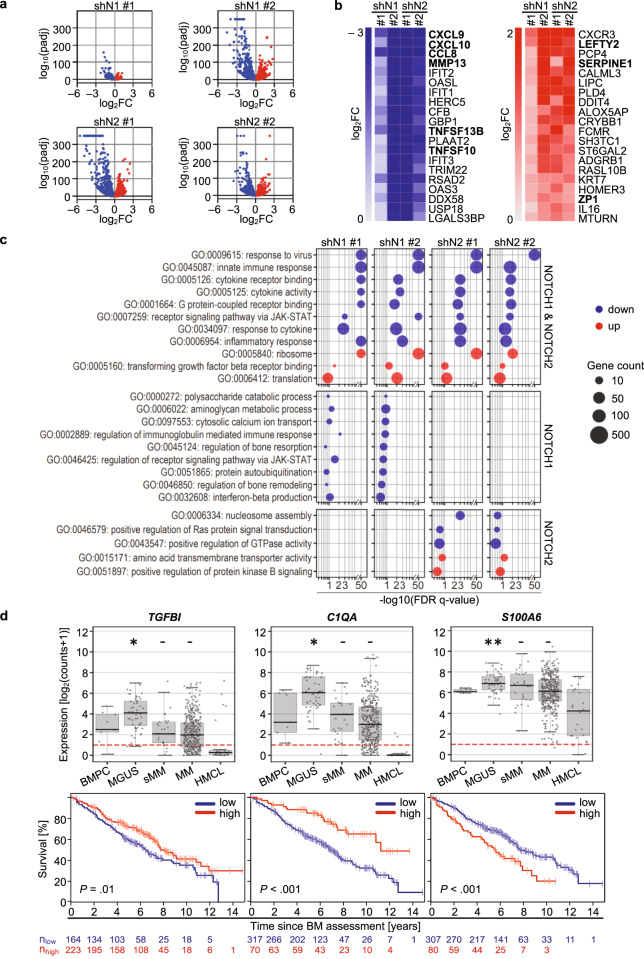
Deregulation of core matrisome and matrisome-associated genes in RPMI 8226 cells after N1 and N2 knockdown and in human primary MM cells with prognostic significance. **a** Volcano plots illustrating the down- (blue) and upregulated (red) genes after N1 (shN1 #1, #2) and N2 (shN2 #1, #2) knockdown in RPMI 8226 cells (padj < 0.01; log_2_ fold change (FC)). **b** Heatmaps showing the 20 most strongly down- (padj < 0.01; log_2_FC < 0) and upregulated (padj < 0.01; log_2_FC > 0) genes. ECM-associated genes in bold. **c** Panel shows GSEA of genes downregulated after N1 and N2 knockdown (shN1 #1, #2; shN2 #1, #2). FDR *Q*-values (<0.25) of gene sets are shown including cytokine activity (N1, N2), and regulation of bone resorption and remodeling (N1). **d** Gene expression of *TGFBI*, *C1QA*, *S100A6* in healthy donor bone marrow plasma cells (BMPC), monoclonal gammopathy of undetermined significance (MGUS), smoldering MM (sMM), untreated MM, and human myeloma cell lines (HMCL). *TGFBI*, *C1QA*, and *S100A6* are differentially expressed in MGUS compared to BMPC. Survival analysis of *TGFBI*, *C1QA*, *S100A6* of patient outcome from the 387 cohort [9]. *P-value ≤ 0.05, **P-value ≤ 0.01.

We transduced human RPMI 8226 and MM.1S cells with shRNAs for knockdown of NOTCH(N)1 and N2 receptors. Efficiency and specificity of depletion were validated by qPCR, flow cytometry analysis, and immunoblotting (Supplementary Fig. 1). RPMI 8226 and MM.1S cells showed different levels of the intracellular cleaved domain of N2 (N2IC), indicating a variable strength of N2 activation (Supplementary Fig. 2). In addition, N1 and N2 depleted MM cells were less viable and more sensitive to bortezomib, melphalan, and lenalidomide (Supplementary Fig. 1), confirming that these receptors control growth and drug resistance. High-throughput transcriptome profiling revealed decreased NOTCH target gene expression of *HES*4, *HES7* in RPMI 8226, and *HEY*2, *HEYL* in MM.1S cells (Supplementary Table 1). Many of the 19,720 analyzed genes were significantly up- or downregulated by at least one of the shRNAs in RPMI 8226 cells (shN1: 2761 up and 2758 down; shN2: 3028 up and 3355 down; cut-off: padj < 0.01), whereas in MM.1S cells less genes were regulated (shN1: 503 up and 1032 down; shN2: 823 up and 1641 down; cut-off: padj < 0.01, Supplementary Table 1, Supplementary Fig. 3). Among the top 20 genes commonly regulated after N1 and N2 depletion, we identified nine matrisome-associated genes in RPMI 8226 cells: (i) down - *CXCL9*, *CXCL10*, *CCL8*, *MMP13, TNFSF13B*, *TNFSF10*, and (ii) up - *LEFTY2*, *SERPINE1*, *ZP1* (Fig. 1). In MM.1S cells, two out of three commonly upregulated genes (*CLEC7A, TGFA*) encode matrisome-associated proteins (Supplementary Fig. 3).

These findings prompted the systematic search for NOTCH-driven matrisome genes within the entire gene expression data set. To this end, we used the MatrisomeDB database that provides live cross-referencing to gene and protein databases for every ECM and ECM-associated gene [1]. GO analysis revealed that N1 and N2 regulate both core matrisome genes and a series of matrisome-associated genes in RPMI 8226 (Table 1) and MM.1S cells (Supplementary Table 2). Overall comparison between up- or downregulated genes showed that expression of 14 and 34 matrisome genes is commonly regulated by N1 and N2 in both cell lines (Supplementary Table 1). QPCR analysis or immunoblotting demonstrated that lower levels of N1 or N2 correlate with lower levels of HPSE in NOTCH-depleted RPMI 8226 and MM.1S cells (Supplementary Fig. 2). The same trend of low HPSE expression can be found in the MM cell line AMO-1, which is characterized by low NOTCH levels. Similarly, lower levels of MMP13, S100A6, IGF1 correspond to decreased NOTCH levels in RPMI 8226 cells, and low levels of MMP13 to low NOTCH levels in AMO-1 cells. In contrast, lower levels of N1 and N2 are associated with higher protein levels of EMID1, TGFBI, and C1QA in RPMI 8226 cells (Supplementary Fig. 2). These data confirm the reliability of the transcriptome analysis and the regulation of matrisome genes through N1 and N2 in MM cells.

**Table 1:** Expression of matrisome genes in RPMI 8226 cells after N1 and N2 knockdown.

		Division	Category	Gene symbol	Gene name	shRNA#1 [log_2_FC]	shRNA#2 [log_2_FC]
N1 knockdown	Genes up	Cor matrisome	ECM glycoproteins	*IGFBP2*	Insulin-like growth factor binding protein 2, 36 kDa	0.705	0.741
				*ZP1*	Zona pellucida glycoprotein 1 (sperm receptor)	0.543	1.017
		Matrisome-associated	ECM-affiliated proteins	*LEFTY2*	Left-right determination factor 2	0.97	1.853
				*PDGFB*	Platelet-derived growth factor beta polypeptide (simian sarcoma viral (v-sis) oncogene homolog)	0.581	0.786
	Genes down	Core matrisome	ECM glycoproteins	*IGFBP4*	Insulin-like growth factor binding protein 4	−1.364	−2.331
				*SPP1*	Secreted phosphoprotein 1	−1.630	−1.471
			Proteoglycans	*HAPLN3*	Hyaluronan and proteoglycan link protein 3	−0.645	−1.355
		Matrisome-associated	ECM-affiliated proteins	*MUC1*	Mucin 1, cell surface associated	−0.563	−0.524
			ECM regulators	*MMP13*	Matrix metallopeptidase 13 (collagenase 3)	−0.824	−4.088
			Secreted factors	*CCL2*	Chemokine (C-C motif) ligand 2	−1.423	−1.950
				*CCL3*	Chemokine (C-C motif) ligand 3	−0.837	−1.722
				*CCL5*	Chemokine (C-C motif) ligand 5	−0.960	−2.597
				*CCL8*	Chemokine (C-C motif) ligand 8	−2.274	−3.680
				*CXCL10*	Chemokine (C-X-C motif) ligand 10	−1.005	−4.661
				*CXCL9*	Chemokine (C-X-C motif) ligand 9	−1.969	−4.213
				*IGF1*	Insulin-like growth factor 1 (somatomedin C)	−0.500	−1.089
				*IL10*	Interleukin 10	−1.451	−1.910
				*TNFSF10*	Tumor necrosis factor (ligand) superfamily, member 10	−0.697	−3.138
				*TNFSF13B*	Tumor necrosis factor (ligand) superfamily, member 13b	−1.563	−3.153
				*WNT5A*	Wingless-type MMTV integration site family, member 5A	−0.688	−0.553
N2 knockdown	Genes up	Core matrisome	ECM glycoproteins	*EMID1*	EMI domain containing 1	1.199	0.995
				*TGFBI*	Transforming growth factor, beta-induced, 68 kDa	0.729	0.851
				*THBS2*	Thrombospondin 2	0.694	0.795
				*ZP1*	Zona pellucida glycoprotein 1 (sperm receptor)	0.858	0.694
		Matrisome-associated	ECM-affiliated proteins	*C1QA*	Complement component 1, q subcomponent, A chain	0.577	0.770
			ECM regulators	*CTSF*	Cathepsin F	0.940	0.605
				*TMPRSS15*	Protease, serine, 7 (enterokinase)	1.646	0.742
				*SERPINE1*	Serpin peptidase inhibitor, clade E (nexin, plasminogen activator inhibitor type 1), member 1	0.559	2.133
			Secreted factors	*S100A9*	S100 calcium binding protein A9	1.065	0.958
				*TNFSF4*	Tumor necrosis factor (ligand) superfamily, member 4	0.621	0.632
				*WNT5B*	Wingless-type MMTV integration site family, member 5B	0.717	0.529
	Genes down	Core matrisome	ECM glycoproteins	*AGRN*	Agrin	−1.081	−0.714
				*IGFBP3*	Insulin-like growth factor binding protein 3	−0.725	−0.776
				*IGFBP4*	Insulin-like growth factor binding protein 4	−2.309	−2.075
				*LRG1*	Leucine-rich alpha-2-glycoprotein 1	−0.948	−0.621
				*NTNG2*	Netrin G2	−0.774	−0.768
			Collagens	*COL16A1*	Collagen, type XVI, alpha 1	−0.850	−0.507
			Proteoglycans	*HAPLN3*	Hyaluronan and proteoglycan link protein 3	−1.399	−0.67
		Matrisome-associated	ECM-affiliated proteins	*LGALS9*	Lectin, galactoside-binding, soluble, 9	−2.045	−0.932
				*SEMA6D*	Sema domain, transmembrane domain (TM), and cytoplasmic domain, (semaphorin) 6D	−0.557	−0.704
			ECM regulators	*HPSE*	Heparanase	−0.934	−1.146
				*KY*	Kyphoscoliosis peptidase	−0.919	−0.875
				*MMP13*	Matrix metallopeptidase 13 (collagenase 3)	−4.951	−2.381
				*PLOD2*	pProcollagen-lysine, 2-oxoglutarate 5-dioxygenase 2	−0.978	−0.770
				*SERPING1*	Serpin peptidase inhibitor, clade G (C1 inhibitor), member 1	−2.603	−2.389
			Secreted factors	*ANGPTL6*	Angiopoietin-like 6	−1.012	−0.590
				*CCL2*	Chemokine (C-C motif) ligand 2	−2.484	−1.191
				*CCL3*	Chemokine (C-C motif) ligand 3	−2.761	−1.950
				*CCL3L3*	Chemokine (C-C motif) ligand 3-like 3	−1.719	−1.452
				*CCL4*	Chemokine (C-C motif) ligand 4	−1.092	−0.677
				*CCL5*	Chemokine (C-C motif) ligand 5	−2.895	−1.974
				*CCL8*	Chemokine (C-C motif) ligand 8	−3.697	−3.540
				*CXCL10*	Chemokine (C-X-C motif) ligand 10	−5.609	−2.752
				*CXCL11*	Chemokine (C-X-C motif) ligand 11	−2.226	−1.982
				*CXCL12*	Chemokine (C-X-C motif) ligand 12 (stromal cell-derived factor 1)	−1.227	−0.556
				*CXCL9*	Chemokine (C-X-C motif) ligand 9	−4.870	−3.239
				*CXCL8*	Interleukin 8	−0.812	−0.591
				*IL15*	Interleukin 15	−1.021	−0.743
				*IL1RN*	Interleukin 1 receptor antagonist	−1.721	−1.592
				*IL23A*	Interleukin 23, alpha subunit p19	−0.827	−0.527
				*MDK*	Midkine (neurite growth-promoting factor 2)	−0.635	−0.750
				*S100A6*	S100 calcium-binding protein A6	−0.713	−0.577
				*TNFSF10*	Tumor necrosis factor (ligand) superfamily, member 10	−3.623	−2.403
				*TNFSF13B*	Tumor necrosis factor (ligand) superfamily, member 13b	−3.128	−2.314

In parallel, we performed gene set enrichment analysis (GSEA) to determine expression changes in gene sets after N1 and N2 depletion. Both receptors activate immune system-associated and cytokine activity signatures such as cytokine receptor binding and inflammatory response in RPMI 8226 cells (Fig. 1) or leukocyte cell-cell adhesion, toll-like receptor signaling pathway, or chemoattractant activity in MM.1S cells (Supplementary Table 3), confirming that NOTCH controls a cytokine network, defining a supportive TME in MM [6]. Interestingly, N1 regulates genes associated with bone remodeling and resorption in RPMI 8226 (GO:0046850; GO:0045124), and N1 and N2 control genes associated with osteoclast differentiation in MM.1S (GO:0045670) cells including *RUNX2* or *SPP1* (Fig. 1; Supplementary Table 3). RUNX2 and SPP1 (osteopontin) control bone homeostasis in skeletal precursors. In MM, RUNX2 may similarly control ECM-modifying genes such as *MMP13* and *SPP1*, and *RUNX2* expression correlated with severity of the disease [7]. High levels of MM cell-derived MMP13 enhance the osteolytic activity of osteoclasts and correlate with bone lesions in MM patients [8]. Of note, SPP1 is upregulated as part of a prognostic cancer core matrisome signature identified by transcriptomics and proteomics in breast and colon cancer [1].

To correlate expression levels of ECM genes and patient survival, we first analyzed gene expression in samples of BM plasma cells (BMPC) from healthy donors, in patient samples from monoclonal gammopathy of undetermined significance (MGUS), smoldering (sMM), untreated MM, and in samples of human MM cell lines (HMCL) [9]. Next, we determined their association with survival in MM patients (Fig. 1). We focused the analysis on the 64 matrisome genes regulated in RPMI 8226 cells (Table 1), since we found the same classes of ECM glycoproteins, regulators, or secreted factors regulated in MM.1 S cells (Supplementary Table 2). Seven out of the 64 matrisome genes, *TGFBI*, *C1QA*, *S100A6, IGF1, HPSE*, *CXCL12*, and *CXCL8*, showed an association with progression-free and overall survival (Fig. 1, Supplementary Fig. 4) in a previously published cohort of MM patients (*n* = 387) [9]. *TGFBI* is an N2-driven target gene with low expression being associated with adverse overall survival (Fig. 1). Accordingly, a global DNA hypermethylation analysis linked the methylation status of *TGFBI* to an unfavorable prognosis [10]. We further identified *C1QA* as a novel N2-regulated ECM gene. High levels of *C1QA* were associated with better prognosis of MM patients (Fig. 1). *C1QA* encodes the A-chain polypeptide of serum complement subcomponent C1q binding to immunoglobulins complexed to antigen and initiating the complement cascade [11]. In skin cutaneous melanoma, C1QA is a novel prognostic biomarker that has a function as a core TME-related gene [11]. Similarly, high levels of NOTCH-driven EMI domain containing 1 (EMID1) correlate with tumor-infiltrating immune cells and are associated with a favorable prognosis in lung adenocarcinoma [12]. S100A6 is a Ca^2+^-binding protein that belongs to the S100 family controlling cell growth, differentiation, and survival in cancer and cancer stem cells [13]. S100A6 binds to ECM-associated proteins such as LUM, PRELP, IGFBP-1, and high serum levels are positively correlated with cancer progression of gastric, non-small cell lung, ovarian, and urinary bladder cancer [13]. We showed that *S100A6* is downregulated after N2 knockdown in MM cells, and high levels were associated with adverse prognosis of MM patients (Fig. 1). Moreover, S100 proteins are classical binding partners of ANXA2, and in pancreatic cancer the interaction between S1006 and ANXA2 promotes motility and invasiveness of cancer cells [14]. In addition, N2 controls the expression of the ECM regulator HPSE that cleaves heparan sulfate glycosaminoglycans from proteoglycan core proteins to small oligosaccharides [15]. HPSE promotes shedding of syndecan-1 from the MM cell surface, modulates the expression of proteases, alters histone acetylation and gene expression, and promotes tumor growth, angiogenesis, and metastasis of MM cells [15].

Taken together, our data show that both NOTCH receptors participate in the transcriptional control of ECM glycoproteins (TGFBI), ECM-affiliated proteins (C1QA), ECM regulators (HPSE) and secreted factors (S100A6, IGF1) in MM cells in vitro, proofing to be of prognostic significance in clinical settings. Our data confirm that the TME and ECM represented by a tumor-associated matrisome contain potential biomarkers and support findings in omental metastasis of ovarian cancer, in which a 22-matrisome gene and protein signature has been identified, predicting overall survival in solid cancers such as breast, head, and neck squamous cell carcinoma, non-small-cell lung adenoma, kidney clear cell carcinoma, hepatocellular carcinoma, colon cancer or pancreatic ductal adenocarcinoma [1]. In MM, similar signatures with prognostic significance should be refined and may confer impact in diagnostic/prognostic classification and the characterization of therapeutic targets as in colorectal cancer [1]. However, further studies are required to comprehensively answer the question how expression changes in NOTCH-driven matrisome-associated proteins in the BM niche promote MM growth and dissemination.

### Supplementary information

Supplemental Material

Supplemental Table 1

Supplemental Table 2

Supplemental Table 3
