# Supplementary material for: Identification of NOTCH-driven matrisome-associated genes as prognostic indicators of multiple myeloma patient survival
Source: Blood Cancer J. 2023 Sep 5;13(1):134. doi: 10.1038/s41408-023-00907-6 (PMC10480158; doi:10.1038/s41408-023-00907-6)
Supplement: Supplementary file 1 — Supplemental Material [file 41408_2023_907_MOESM1_ESM.docx]

**Supplementary information**

Identification of NOTCH-driven matrisome-associated genes as prognostic indicators of multiple myeloma patient survival

Daniela Simone Maichl, Julius Arthur Kirner, Susanne Beck, Wen-Hui Cheng, Melanie Krug, Martin Kuric, Carsten Patrick Ade, Thorsten Bischler, Franz Jakob, Dirk Hose, Anja Seckinger, Regina Ebert, and Franziska Jundt

**Supplementary Materials and Methods**

**Cell culture**

MM.1S and RPMI 8226 cell lines were cultured under standard conditions in 5 % CO_2_ at 37 °C in RPMI 1640 medium with GlutaMAX (Thermo Fisher Scientific, Darmstadt, Germany), supplemented with 10 % fetal calf serum (FCS; Anprotec, Bruckberg, Germany), 1 % penicillin/streptomycin (10.000 U/ml, Thermo Fisher Scientific, Darmstadt, Germany), and 1 % sodium pyruvate (100 mM, Thermo Fisher Scientific). Medium of transduced cells was further supplemented with 0.75 µg/ml puromycin (Thermo Fisher Scientific). HEK293T cells were cultured in Dulbecco’s modified Eagle’s medium (Thermo Fisher Scientific) containing 10 % FCS, 1 % penicillin/streptomycin, and 1 % sodium pyruvate. Penicillin/streptomycin-free medium additionally supplemented with a 10 mM HEPES buffer (Sigma-Aldrich GmbH, Steinheim, Germany) was used during production of lentivirus.

**Cloning**

Two specific shRNA sequences against NOTCH1 (shN1 #1, shN1 #2) and NOTCH2 (shN2 #1, shN2 #2) were obtained from the literature.^1^ The 97-mer oligonucleotides (Supplementary Table 4) as well as a non-targeting control (NTC) shRNA were amplified by PCR and cloned into the lentiviral expression vector pGIPZ (Dharmacon, Lafayette, CO, USA) at the EcoRI and XhoI restriction sites. Plasmid sequences were verified by commercial sequencing (LGC Genomics, Berlin, Germany) using a pGIPZ-specific sequencing primer (5'‑GCATTAAAGCAGCGTATC-3').

**Lentivirus production and transduction**

Lentivirus were produced in HEK293T cells through co-transfection with a mix of 10 µg transfer plasmids, 5 µg packaging plasmid psPAX.2 (#12260, Addgene, Watertown, MA, USA), and 2.5 µg envelope plasmid pMD2.G (#12259, Addgene) using the TransIT-Lenti transfection reagent (Mirus, Madison, WI, USA) according to the manufacturer’s protocol. After incubating the transfected cells in an incubator at 37 °C for 48 h, the virus-containing supernatant was harvested and filtered through a 0.45 µm filter. Virus supernatant was immediately used for transduction or shock frozen with liquid nitrogen and stored at -80 °C until further use. Cells were transduced by spinoculation. Therefore, cells were harvested and resuspended in medium at a density of 10^6^ cells/ml. Then, 1 ml cell suspension was mixed with 1 ml virus supernatant and polybrene (8 µg/ml final concentration) and transferred into 15 ml reaction tubes. The mix was centrifuged at 800 g at 32 °C for 1.5 h. Afterwards, cells were washed with cell culture medium two times. Selection started after 24h by adding puromycin.

**Flow cytometry**

NOTCH1 and NOTCH2 expression was analyzed by intracellular staining and flow cytometry. Therefore, cells were washed with washing solution (PBS containing 5 % FCS), fixed using 4 % paraformaldehyde (PFA) in PBS and permeabilized with 90 % methanol on ice for 30 min. After incubation with anti-NOTCH1 or anti-NOTCH2 primary antibodies (1:800 in washing solution; Supplementary Table 5) for 1 h at RT, cells were washed and incubated with the secondary antibody (1:500 in washing solution, Supplementary Table 5) for 1 h at RT. Cells were then washed again and resuspended in PBS. Flow cytometric data was acquired on a FACSCanto™ II (BD Biosciences, Heidelberg, Germany) and analyzed using the software Flowing v2.5.1 (Cell Imaging Core, Turku Center for Biotechnology, Turku, Finland).

**Immunoblotting**

For preparing whole cell lysates, cells were lysed using NP-40 lysis buffer (50 mM Tris pH 7.5, 150 mM NaCl, 1 % Triton, 0.5 % NP-40, and 0.2 % SDS). Subsequently, protein was quantified using the Pierce^TM^ BCA Protein Assay Kit (Thermo Fisher Scientific) following the manufacturer’s instruction or Roti®-Quant reagent (Carl Roth GmbH, Karlsruhe, Germany), as previously described.^2^ Samples were prepared by mixing 50 µg protein with Laemmli buffer (50 mM Tris-HCl pH 6.8, 1 % SDS, 10 % glycerol, 0.02 % bromophenol blue, 1 % ß-Mercaptoethanol) and boiling at 95 °C for 5 min. Proteins were separated by sodium dodecyl sulfate-polyacrylamide gel electrophoresis (SDS-PAGE) on 10 % or 12 % polyacrylamide gels and transferred to a Protran® nitrocellulose membrane (Whatman, Dassel, Germany) at 1.5 mA/cm^2^ of the membrane area for 2 h. The membrane was blocked with 5 % bovine serum albumin (BSA) or 5% non-fat skim milk in TBS-T buffer (50 mM Tris, 150 mM NaCl, 0.1 % Tween-20) for 2 h and then incubated with the primary antibodies (1:1,000 in 5 % BSA/TBS-T) overnight at 4 °C. The membrane was washed three times with TBS-T, followed by incubation with the secondary antibody (1:10,000 in TBS-T) for 45 min at RT, prior to another washing with TBS-T and rinsing with ddH_2_O. Proteins were detected using the SuperSignal™ West Dura Extended Duration Substrate (Thermo Fisher Scientific) and a ChemiDoc MP Imaging System (Bio-Rad Laboratories, Hercules, CA, USA). Afterwards, the membrane was washed again with TBS-T and stripped to remove antibodies by incubation in stripping buffer (1.5 % glycine, 0.1 % SDS, 1 % Tween-20, pH 2.2) for 30 min at RT, shaking. Subsequently, the membrane was again blocked and re-stained with the loading control, as described above. GAPDH (1:1,000 or 1: 5,000 in TBS-T) was used as loading control.

**Quantitative PCR**

For qPCR analysis in Supplementary Figure 2, total RNA was extracted with the RNeasy Plus Mini kit (Qiagen, Hilden, Germany) following the manufacturer's protocol. Concentrations of the RNA extracts were measured using a Multiskan™ GO microplate spectrophotometer (Thermo Fisher Scientific). For reverse transcription, 2 µg RNA was used for each sample. First strand cDNA synthesis was performed using SuperScript™ III Reverse Transcriptase (Thermo Fisher Scientific) in a total volume of 20 µl. Samples were then diluted by adding 80 µl nuclease-free water. For each 10 µl quantitative PCR (qPCR) reaction, 1 µl of diluted cDNA was used. The qPCR reactions were performed in technical triplicates and by using the SYBR™ Select Master Mix for CFX (Thermo Fisher Scientific) according to the manufacturer’s protocol in a StepOnePlus™ Real-Time PCR System (Applied Biosystems, Waltham, MA, USA). Log_2_ fold changes (log_2_FC) were calculated with the ΔΔCt-method using the StepOne‑Plus Software (Applied Biosystems). *HPRT* was used as housekeeping gene.

For qPCR analysis in Supplementary Figure 3, total RNA was extracted with the NucleoSpin RNA II kit (Macherey-Nagel, Düren, Germany) following the manufacturer's protocol. Concentrations of the RNA extracts were measured using a BioPhotometer® D30 (Eppendorf SE, Hamburg, Germany). For reverse transcription, 1 µg RNA was used for each sample. First strand cDNA synthesis was performed using M-MLV Reverse Transcriptase, RNase H Minus, Point Mutant (Promega, Mannheim, Germany) in a total volume of 25 µl. Samples were then diluted by adding 225 µl nuclease-free water. For each 20 µl quantitative PCR (qPCR) reaction, 2 µl of diluted cDNA was used. The qPCR reactions were performed in technical triplicates and by using the GoTaq® qPCR Master Mix (Promega) according to the manufacturer’s protocol in a qTOWER3 (Analytik Jena AG, Jena, Germany) Real-Time PCR System followed by melting curve analysis for specificity of qPCR products. Fold changes were calculated with the efficiency-corrected ΔΔCt-method.^3^ 18s and RPS27A were used as housekeeping genes. Primer sequences are shown in Supplementary Table 6.

**CellTiter-Glo Assay**

To investigate the effects of NOTCH1 and NOTCH2 knockdown on viability of MM cell lines, transduced MM.1S and RPMI 8226 cells were seeded in 6-well plates at a density of 3 × 10^5^ or 10^5^ cells/well, respectively. Cells were treated with bortezomib, melphalan, and lenalidomide. After 2 days, 2 ml fresh medium and drugs were added. The relative cell viability was determined after 4 days using the CellTiter-Glo® Luminescent Cell Viability Assay (Promega, Fitchburg, WI, USA) according to the manufacturer‘s protocol. Luminescence of technical triplicates was measured using a Luminoskan Ascent Microplate Luminometer (Thermo Fisher Scientific).

**Cell cycle analysis**

For cell cycle analysis, MM cells were seeded in 12-well plates at the density of 1 × 10^5^ cells/ml. After treatment with bortezomib, melphalan, and lenalidomide for 24 hours, cells were harvested, washed with PBS, fixed in 4 % PFA, and stained with 10 µg/ml DAPI (FxCycle Violet stain, Thermo Fisher Scientific) in 0.1% Triton X-100/PBS. After incubation in the dark at RT for 30 minutes, cell cycle profiles were analyzed using a MACSQuant Analyzer 10 Flow Cytometer (Miltenyi Biotec, Bergisch Gladbach, Germany). The figures are plotted using the software FlowJo (BD Biosciences, Heidelberg, Germany).

**RNA sequencing and bioinformatics analysis**

Total RNA was extracted from 1 × 10^7^ cells of the transduced MM.1S and RPMI 8226 cell lines using the RNeasy Plus Mini kit (Qiagen, Hilden, Germany) according to the manufacturer's protocol. We used the NEBNext Poly(A) mRNA Magnetic Isolation Module (New England Biolabs, Frankfurt, Germany) to remove rRNA from the obtained total RNA extracts. Library preparation was performed using the NEBNext Ultra II RNA Library Prep Kit for Illumina (New England Biolabs) following the manufacturer’s protocol. AMPure XP beads (Beckman Coulter, Beverly, MA, USA) were used to size-select the libraries. Samples were amplified and sample-specific indexes were introduced for the sequencing step using NEBNext Multiplex Oligos for Illumina (Dual Index Primer Set 1, Illumina, New England Biolabs) in an 8-cycle PCR reaction. Library size and concentration of the samples was determined with an Agilent Bioanalyzer 2100 (Thermo Fisher Scientific). Libraries were sequenced with a NextSeq 500 and a MiniSeq System (Illumina, San Diego, CA, USA).

Sequencing reads were quality- and adapter-trimmed via Cutadapt version 2.5,^4^ using a cutoff Phred score of 20 in NextSeq mode, and reads without any remaining bases were discarded (command line parameters: --nextseq-trim=20 -m 1 -a AGATCGGAAGAGCACACGTCTGAACTCCAGTCAC).

Processed reads were subsequently mapped to the human genome (GRCh38.p13 primary assembly and mitochondrion) using STAR v2.7.2b with default parameters based on RefSeq annotation version 109.20200522 for GRCh38.p13.^5^ Read counts on exon level summarized for each gene were generated using featureCounts v1.6.4 from the Subread package.^6^ Multi-mapping and multi-overlapping reads were counted strand-unspecific with a fractional count for each alignment and overlapping feature (command line parameters: -s 0 -t exon -M -O --fraction). The count output was utilized to identify differentially expressed genes using DESeq2 version 1.24.0.^7^ Read counts were normalized by DESeq2 and fold-change shrinkage was applied by setting the parameter “betaPrior=TRUE”. RNA sequencing (RNAseq) data are available at GEO under accession number GSE221038. The strength and consistency of regulation was evaluated for all coding and non-coding RNA. First, an adjusted *P*‑value cutoff (*padj* < 0.01) was applied. All remaining genes were overlapped in Venn diagrams using the online tool Venny 2.1.0.^8^ For the ones overlapping in all four conditions (shN1 #1 vs shNTC, shN1 #2 vs shNTC, shN2 #1 vs shNTC, shN2 #2 vs shNTC) the mean of regulation was calculated for ranking them by their strength of regulation (log_2_FCs). The 20 most strongly upregulated and downregulated genes were included in a heatmap. For gene set enrichment analysis (GSEA) no *P*‑value cutoff was applied. Preranked gene lists were analyzed using the software GSEA v4.1.0 and official gene sets downloaded from the GSEA website (www.broadinstitute.org/gsea/). An arbitrary FDR q-value cutoff of 0.25 was implemented and non-significant GO terms were removed. The remaining terms were overlapped and sorted for regulation through NOTCH1 and NOTCH2 (shN1 #1, shN1 #2, shN2 #1, and shN2 #2), exclusively through NOTCH1 (shN1 #1 and shN1 #2) or exclusively through NOTCH2 (shN2 #1 and shN2 #2). GO terms were ranked by the FDR q-value. Further, the gene count that includes all significantly regulated genes (*padj* < 0.01) was determined for each shRNA for each GO term.

**Expression of targets in human MM cells**

Malignant plasma cells obtained from healthy donor bone marrow plasma cells (BMPC, n=9), MGUS (n=52), smoldering MM (sMM, n=29), untreated MM patients (n=387), and human MM cell lines (HMCL, n=19) were examined by RNAseq and analyzed as previously published.^9^ In brief, RNAseq-based FASTQ-files were analyzed with STAR with default options.^5^ A genome reference was generated with STAR using GRCh38 genome and annotation information of Ensembl databases release 82.^10^ Files were aligned to GRCh38 genome build and reads counted per gene. STAR uses HTSeq^11^ internally for counting reads and sorts the resulting bam files by coordinate. Technical replicates were summed up and reads per gene were normalized with edgeR.^12^ Therefore, normalization factors were calculated with default options and counts per million were computed with accounting for library size. Then log2 transformation was performed with a prior count of 1. This was defined as expression values. Differences of expression of NOTCH-regulated matrix genes (targets) between defined groups were investigated by exact Wilcoxon rank-sum test.^13^ For the analysis of the prognostic value of the targets, MM patients with “high” and “low” target expression were delineated using maximally selected rank statistics (https://cran.r-project.org/web/packages/maxstat/index.html). Overall survival (OS, 387 MM patients) and progression free survival (PFS, 384 MM patients) was analyzed with the Kaplan-Meier method^14^ for high versus low expression of the target gene. Significant differences between the curves were analyzed with log-rank tests.^15^ Analyses were performed with R version 3.6.3.

**Supplementary Figures**

**Supplementary Figure 1
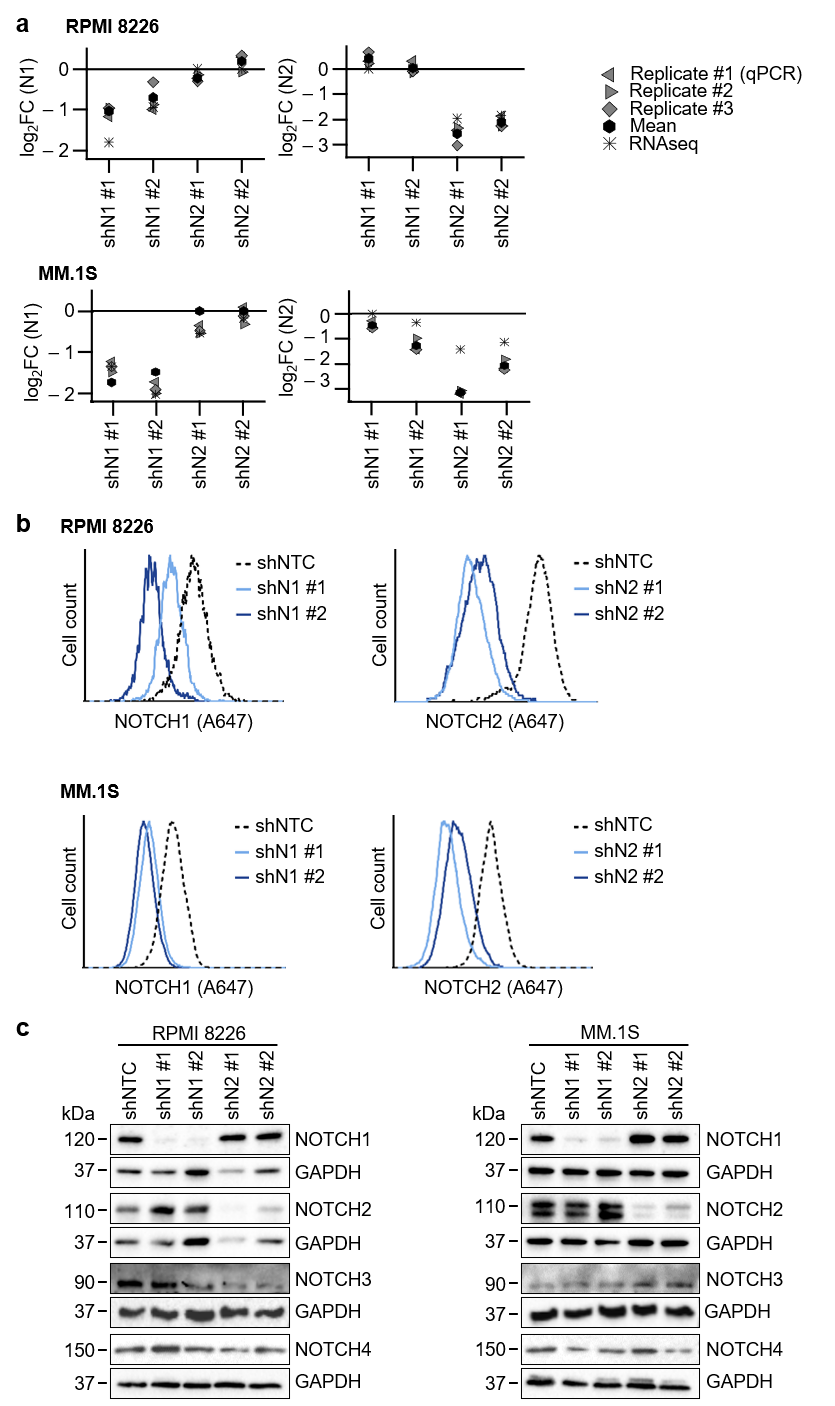
**

| **d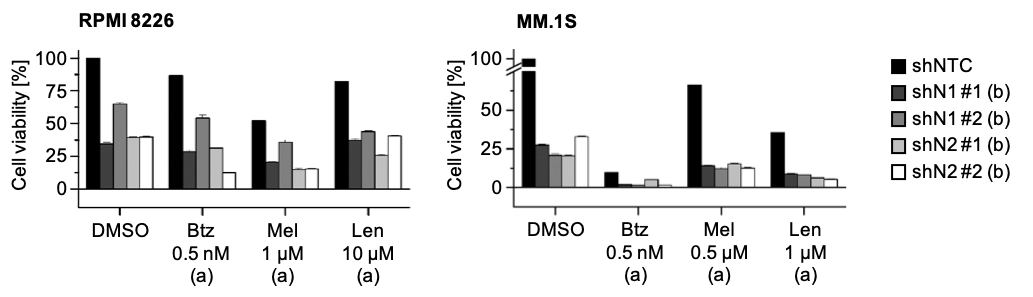** |
| --- |
| **e**  **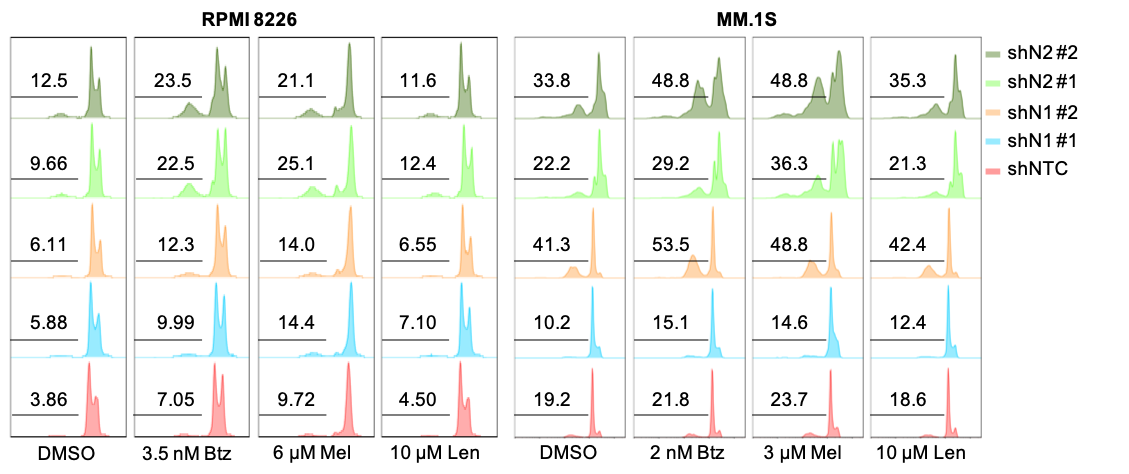** |

**Supplementary Figure 1. Depletion of N1 (shN1 #1, shN1 #2) and N2 (shN2 #1, shN2 #2) in RPMI 8226 and MM.1S cells.** (**a**) Efficacy of N1 and N2 depletion on RNA level by qPCR analysis compared to expression changes determined by RNAseq analysis. (**b**) Depletion of N1 and N2 is confirmed by flow cytometry analysis. (**c**) Efficacy and specificity of N1 and N2 depletion is shown by immunoblotting of whole cell extracts using antibodies against N1, N2, N3, and N4. Representative results are shown for one experiment from three independent experiments. (**d**) Decreased viability in RPMI 8226 (left) and MM.1S (right) cells after N1 and N2 knockdown compared to controls (shNTC) upon treatment with bortezomib, melphalan, and lenalidomide for 4 days. Cell viability was measured using the CellTiter-Glo® Luminescent Cell Viability Assay and plotted relative to DMSO-treated cells transduced with the non-targeting-shRNA. Data is expressed as mean ± SD of one representative experiment. For statistical analysis, two-way ANOVA tests followed by post-hoc Bonferroni were performed. Main effects are shown for drugs (a) and shRNA (b). Effects are significant for *P* < 0.05. (**e**) Increased proportion of apoptotic cells in RPMI 8226 and MM.1S cells after N1 and N2 knockdown and upon treatment with bortezomib, melphalan, and lenalidomide for 48 h and 24 h, respectively. Cell cycle profiles were analyzed by flow cytometry after DAPI staining. Data is shown as the percentage of apoptotic cells from one representative experiment.

**Supplementary Figure 2**

**a**

**
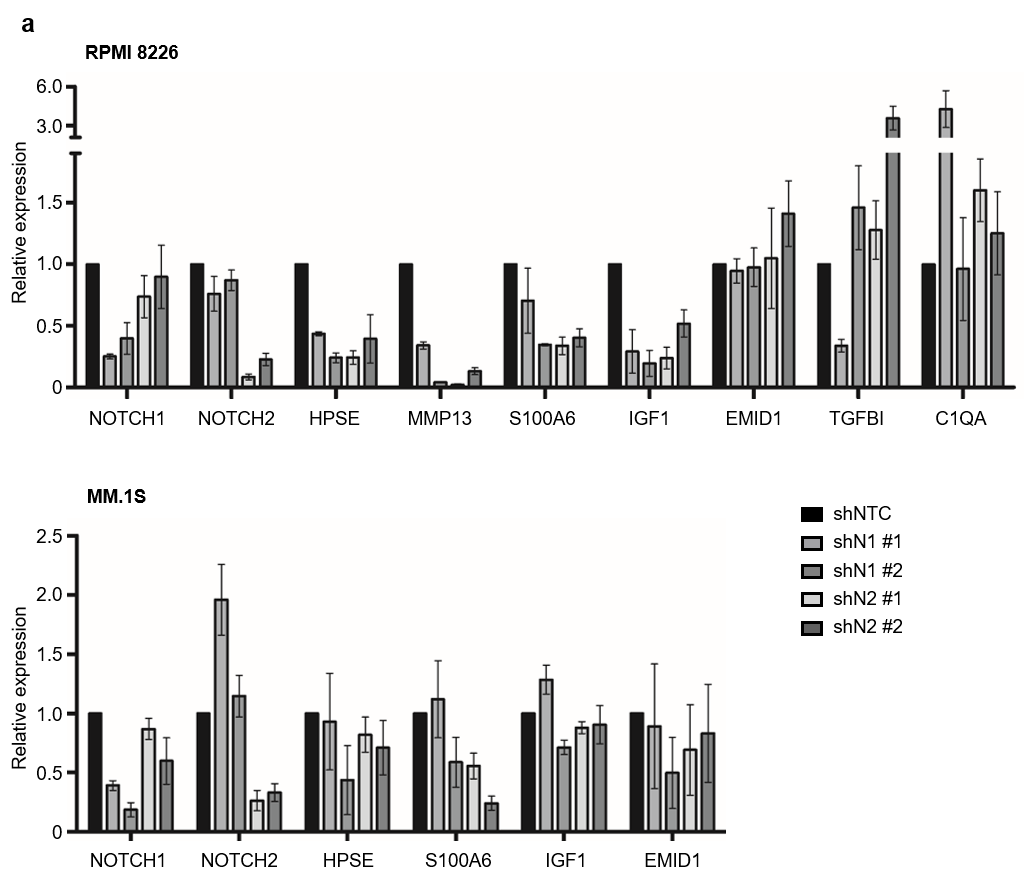
**

| **b** |
| --- |
| **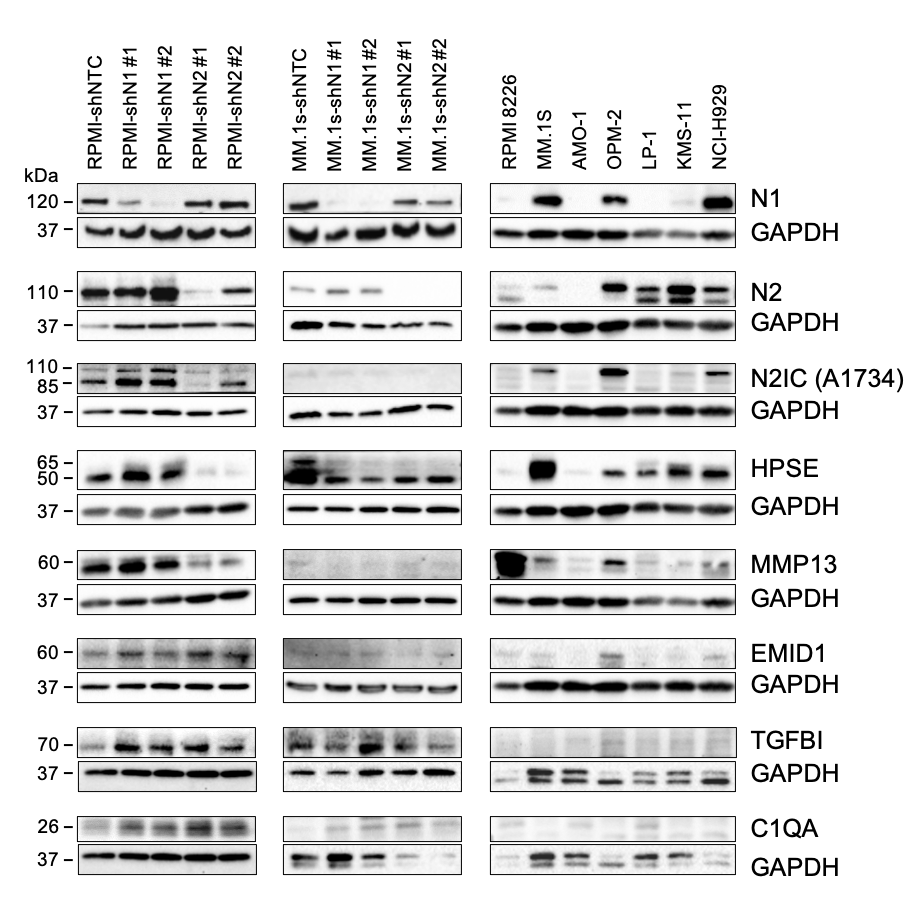** |

**Supplementary Figure 2. Correlation analysis by qPCR and immunoblotting of N1, N2, and selected matrisome factors.** (**a**) QPCR analysis of N1, N2, HPSE, MMP13, S100A6, IGF1, EMID1, TGFBI, C1QA in NOTCH-depleted RPMI 8226, and N1, N2, HPSE, S100A6, IGF1, EMID1 in NOTCH-depleted MM.1S cells. Data is expressed as mean ± SD of one representative experiment. Data were calculated with the ΔΔCt method and normalized to shNTC values by using the housekeeping genes RPS27A (RPMI 8226) and 18s (MM.1S), respectively. (b) Immunoblotting of N1, N2, N2IC, HPSE, MMP13, EMID1, TGFBI, and C1QA in NOTCH-depleted RPMI 8226, MM.1S cells, and in MM cell lines.

**Supplementary Figure 3**

­
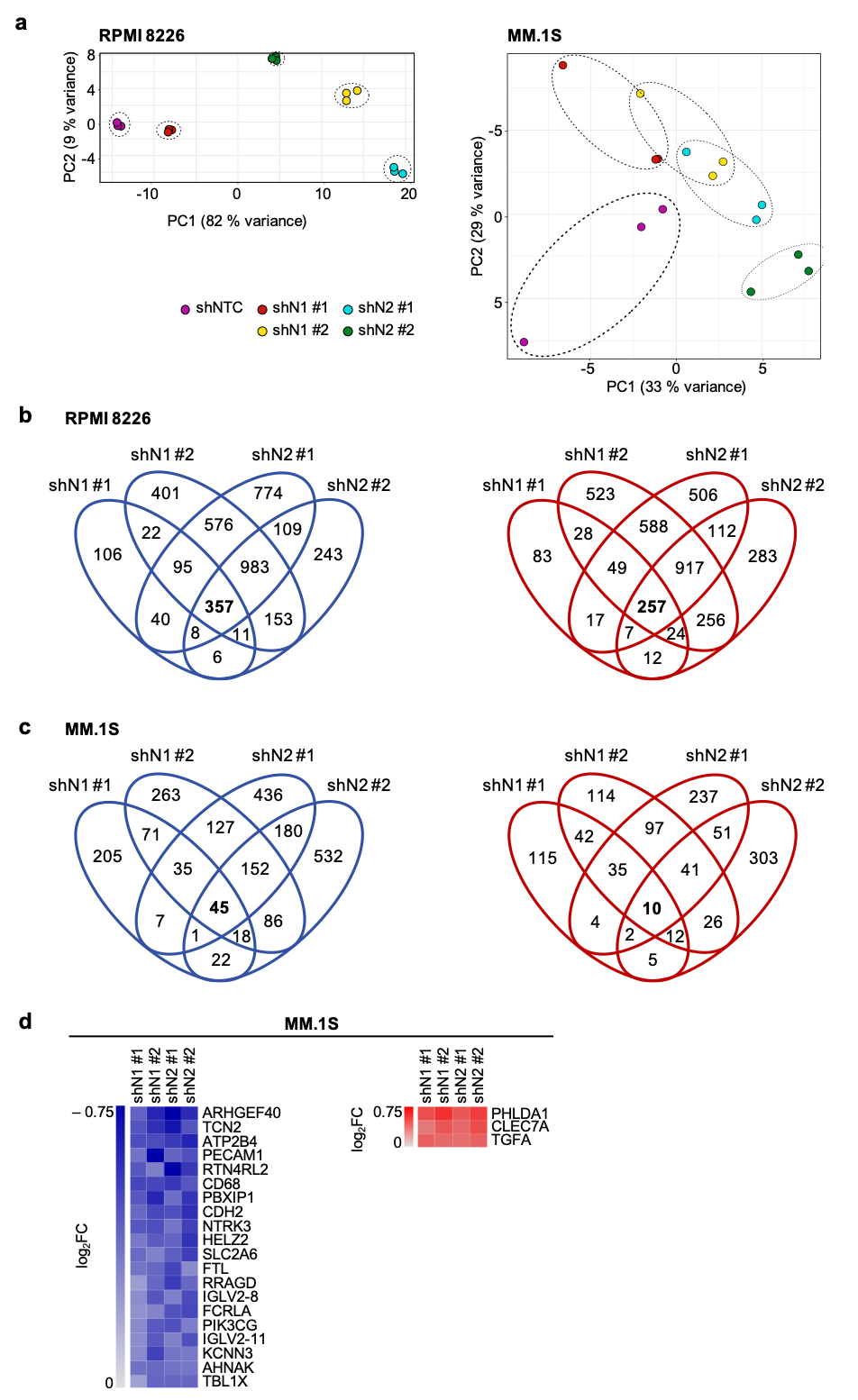


**Supplementary Figure 3. Overlapping genes in RPMI 8226 and MM.1S cells.** (**a**) PCA revealed that the biological replicates of the shRNA transduced cells cluster closely together in RPMI 8226 cells, whereas we observed one outlier of the replicates in MM.1S cells. (**b-c**) Venn diagrams showing the overlap of upregulated (red) and downregulated (blue) genes in RPMI 8226 and MM.1S cells in all four knockdown conditions (shN1 #1, shN1 #2, shN2 #1, shN2 #2). (**d**) Heatmaps showing the 20 most strongly downregulated (*padj* < 0.01; log_2_FC < 0) and the three most strongly upregulated (*padj* < 0.01; log_2_FC > 0) protein-coding genes in MM.1S cells.

**Supplementary Figure 4**

**
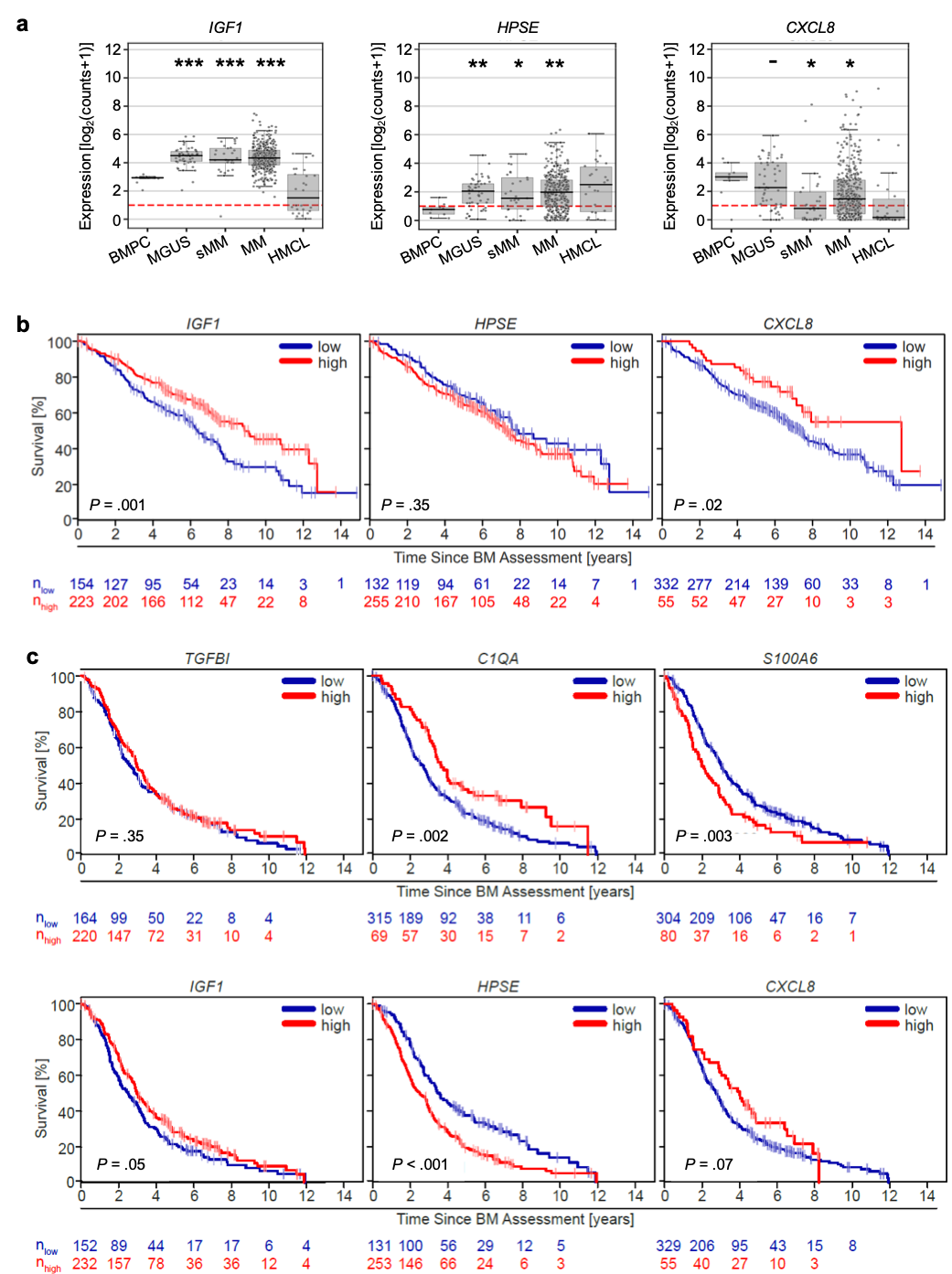
**

**Supplementary Figure 4.** Prognostic significance of deregulated core matrisome and matrisome-associated genes in MM cells. (**a**) Gene expression of *IGF1, HPSE*, and *CXCL8* in normal bone marrow plasma cells (BMPC), MGUS, smoldering MM (sMM), MM, and human MM cell lines (HMCL). Genes are differentially expressed in MGUS, sMM, and MM compared to BMPC. (**b**) Survival analysis (overall survival) of *IGF1, HPSE*, *CXCL8* of patient outcome data from the 387 cohort. (**c**) Survival analysis (progression-free survival) of *TGFBI, C1QA, S100A6*, *IGF1, HPSE*, and *CXCL8* of patient outcome data from the 384 cohort. **P*-value ≤ 0.05, ***P*-value ≤ 0.01, ****P*-value ≤ 0.001.

**Supplementary Table 1. Differential expression of NOTCH target genes after NOTCH knockdown.**

For details see Supplementary excel sheet. Log_2_FC values of all genes regulated by at least one shRNA compared to shNTC (shN1 #1 vs shNTC; shN1 #2 vs shNTC; shN2 #1 vs shNTC; shN2 #2 vs shNTC) are shown in the sheet tabs RPMI 8226_1x sig only and MM.1S_1x sig only. Log_2_FC values of the genes commonly regulated by at least one shRNA compared to shNTC in both cell lines is shown in the sheet tabs RPMI 8226_MM.1S_N1 or RPMI 8226_MM.1S_N2. Core matrisome and matrisome-associated genes are marked in yellow. Fourteen N1-regulated and 34 N2-regulated genes were identified, indicating that ECM genes are commonly regulated by N1 and N2 in MM cells. Some of the *P*-values are set to NA. Reasons are given in the reference analyzing RNAseq data with DESeq2.^7^ Gray shading of boxes, if *P*-values (*padj > 0.01*) were non-significant.

**Supplementary Table 2. Matrisome genes identified by transcriptome profiling in RPMI 8226 and MM.1S cells after N1 and N2 knockdown**

For details see Supplementary excel sheet. Gray shading of boxes, if *P*-values (padj > 0.01) were non-significant.

**Supplementary Table 3. Gene set enrichment analysis of MM.1S cells after NOTCH1 and NOTCH2 knockdown.**

For details see Supplementary excel sheet.

**Supplementary Table 4. shRNA sequences for knockdown of NOTCH1 and NOTCH2 in RPMI 8226 and MM.1S cells.**

| **shRNA** | **shRNA ID** | **Ordered 97-mer sequence** | **Reference** |
| --- | --- | --- | --- |
| shN1 #1 | NOTCH1_4851_7986 | TGCTGTTGACAGTGAGCGCACAAAGATTCATGATTTATAATAGTGAAGCCACAGATGTATTATAAATCATGAATCTTTGTTTGCCTACTGCCTCGGA | Pelossof et al. 2017, Supplement Table S3 |
| shN1 #2 | NOTCH1_4851_7767 | TGCTGTTGACAGTGAGCGAACATGTTTTTATACAAAATAATAGTGAAGCCACAGATGTATTATTTTGTATAAAAACATGTGTGCCTACTGCCTCGGA | Pelossof et al. 2017, Supplement Table S3 |
| shN2 #1 | NOTCH2_4853_2669 | TGCTGTTGACAGTGAGCGATGCCAGGTGAATATTGATGAATAGTGAAGCCACAGATGTATTCATCAATATTCACCTGGCAGTGCCTACTGCCTCGGA | Pelossof et al. 2017, Supplement Table S3 |
| shN2 #2 | NOTCH2_4853_1883 | TGCTGTTGACAGTGAGCGACAGATTGATATTGATGACTGTTAGTGAAGCCACAGATGTAACAGTCATCAATATCAATCTGGTGCCTACTGCCTCGGA | Pelossof et al. 2017, Supplement Table S3 |

**Supplementary Table 5. Antibodies.**

| **Antibodies** | **Sources** | **Catalog #** | **Applications** |
| --- | --- | --- | --- |
| NOTCH1 | Cell Signaling Technology | 4380 | IB, FC |
| NOTCH2 | Cell Signaling Technology | 5732 | IB, FC |
| N2IC (A1734) | Thermo Fisher Scientific | PA5-37433 | IB |
| NOTCH3 | Cell Signaling Technology | 5276 | IB |
| NOTCH4 | Santa Cruz Biosciences | sc-393893 | IB |
| HPSE | Cell Signaling Technology | 99756 | IB |
| MMP13 | Cell Signaling Technology | 69926 | IB |
| EMID1 | Thermo Fisher Scientific | PA5-51418 | IB |
| TGFBI | Cell Signaling Technology | 5601 | IB |
| C1QA | Thermo Fisher Scientific | **MA5-31661** | IB |
| GAPDH | Cell Signaling Technology | 5174 | IB |
| Rabbit IgG-Alexa Fluor 647 | Thermo Fisher Scientific | A-21245 | FC (2^nd^ Ab) |
| Rabbit IgG-HRP | Cell Signaling Technology | 7074 | IB (2^nd^ Ab) |
| Mouse IgG, HRP | Cell Signaling Technology | 7076 | IB (2^nd^ Ab) |

IB, immunoblotting; FC, flow cytometry; 2^nd^ Ab, secondary antibody

**Supplementary Table 6. Primers for qPCR.**

| **Target genes** | **Directions** | **Sequences (5’ to 3’)** |
| --- | --- | --- |
| *NOTCH1* | Forward | ACGCACAAGGTGTCTTCCA |
| *NOTCH1* | Reverse | AGGATCAGTGGCGTCGTG |
| *NOTCH2* | Forward | tggtggcagaactgatcaac |
| *NOTCH2* | Reverse | ctgcccagtgaagagcagat |
| *NOTCH3* | Forward | GCATTCCTTTGTGTCCCTGT |
| *NOTCH3* | Reverse | GGCTTGGGAATTCAGCTACA |
| *NOTCH4* | Forward | TTCCACTGTCCTCCTGCCAGAA |
| *NOTCH4* | Reverse | TGGCACAGGCTGCCTTGGAATC |
| *HPRT* | Forward | CTGGCGTCGTGATTAGTGAT |
| *HPRT* | Reverse | CTCGAGCAAGACGTTCAGTC |
| *18sRNA* | Forward | GTAACCCGTTGAACCCCATT |
| *18sRNA* | Reverse | CCATCCAATCGGTAGTAGCG |
| *RPS27A* | Forward | TCGTGGTGGTGCTAAGAAAA |
| *RPS27A* | Reverse | TCTCGACGAAGGCGACTAAT |
| *HPSE* | Forward | CGGACTGCTACCAAGGAAGA |
| *HPSE* | Reverse | GCCTCCATATGCAGAGCTTG |
| *MMP13* | Forward | TGCCATTACCAGTCTCCGAG |
| *MMP13* | Reverse | TGCAGCATCAATACGGTTGG |
| *EMID1* | Forward | GGTTGTCTCAACTGCAGCAA |
| *EMID1* | Reverse | GGTAGCTGGTGTTGGAGGTA |
| *TGFBI* | Forward | GGACATGCTCACTATCAACGGG |
| *TGFBI* | Reverse | CTGTGGACACATCAGACTCTGC |
| *C1QA* | Forward | CCAGGAAGAACCGTACCAGA |
| *C1QA* | Reverse | GACGATGGACAGGCAGATTT |
| *S100A6* | Forward | TCTTCCACAAGTACTCCGGC |
| *S100A6* | Reverse | TCCGGTCCAAGTCTTCCATC |
| *IGF1* | Forward | TGGATGCTCTTCAGTTCGTG |
| *IGF1* | Reverse | CTGACTTGGCAGGCTTGAG |

**References**

1. Pelossof R, Fairchild L, Huang CH, Widmer C, Sreedharan VT, Sinha N*, et al.* Prediction of potent shRNAs with a sequential classification algorithm. *Nat Biotechnol* 2017 Apr; **35**(4)**:** 350-353.

2. Jundt F, Anagnostopoulos I, Forster R, Mathas S, Stein H, Dorken B. Activated Notch1 signaling promotes tumor cell proliferation and survival in Hodgkin and anaplastic large cell lymphoma. *Blood* 2002 May 1; **99**(9)**:** 3398-3403.

3. Pfaffl MW. A new mathematical model for relative quantification in real-time RT-PCR. *Nucleic Acids Res* 2001 May 1; **29**(9)**:** e45.

4. Martin M. Cutadapt Removes Adapter Sequences From High-Throughput Sequencing Reads. *EMBnetjournal* 2011 May; **17**(1)**:** 10-12.

5. Dobin A, Davis CA, Schlesinger F, Drenkow J, Zaleski C, Jha S*, et al.* STAR: ultrafast universal RNA-seq aligner. *Bioinformatics* 2013 Jan 1; **29**(1)**:** 15-21.

6. Liao Y, Smyth GK, Shi W. featureCounts: an efficient general purpose program for assigning sequence reads to genomic features. *Bioinformatics* 2014 Apr 1; **30**(7)**:** 923-930.

7. Love MI, Huber W, Anders S. Moderated estimation of fold change and dispersion for RNA-seq data with DESeq2. *Genome Biol* 2014; **15**(12)**:** 550.

8. Oliveros JC. An Interactive Tool for Comparing Lists with Venn’s Diagrams. <https://bioinfogp.cnb.csic.es/tools/venny/index.html>. 2007-2015.

9. Seckinger A, Hillengass J, Emde M, Beck S, Kimmich C, Dittrich T*, et al.* CD38 as Immunotherapeutic Target in Light Chain Amyloidosis and Multiple Myeloma-Association With Molecular Entities, Risk, Survival, and Mechanisms of Upfront Resistance. *Front Immunol* 2018; **9:** 1676.

10. Kersey PJ, Allen JE, Armean I, Boddu S, Bolt BJ, Carvalho-Silva D*, et al.* Ensembl Genomes 2016: more genomes, more complexity. *Nucleic Acids Res* 2016 Jan 4; **44**(D1)**:** D574-580.

11. Anders S, Pyl PT, Huber W. HTSeq--a Python framework to work with high-throughput sequencing data. *Bioinformatics* 2015 Jan 15; **31**(2)**:** 166-169.

12. Robinson MD, McCarthy DJ, Smyth GK. edgeR: a Bioconductor package for differential expression analysis of digital gene expression data. *Bioinformatics* 2010 Jan 1; **26**(1)**:** 139-140.

13. Bauer DF. Constructing Confidence Sets Using Rank Statistics. *Journal of the American Statistical Association* 1972 1972/09/01; **67**(339)**:** 687-690.

14. Kaplan EL, Meier P. Nonparametric Estimation from Incomplete Observations. *Journal of the American Statistical Association* 1958 1958/06/01; **53**(282)**:** 457-481.

15. Harrington DP, Flemming TR. A class of rank test procedures for censored survival data. *Biometrika* 1982; **69**(3)**:** 553-566.
